# Percutaneous epiphysiodesis using transphyseal screws for limb-length discrepancies: high variability among growth predictor models

**DOI:** 10.1007/s11832-015-0687-3

**Published:** 2015-09-30

**Authors:** Bryan C. Monier, David D. Aronsson, Michael Sun

**Affiliations:** Department of Orthopaedics and Rehabilitation, University of Vermont College of Medicine, Robert T. Stafford Hall, 4th Floor, 95 Carrigan Drive, Burlington, VT 05405-0084 USA; University of Vermont College of Medicine, Office of the Dean, E-126 Given Building, 89 Beaumont Ave, Burlington, VT 05405-0068 USA

**Keywords:** Limb-length discrepancy, Percutaneous epiphysiodesis using transphyseal screws (PETS), Green–Anderson growth remaining method, Moseley graph method, Paley multiplier method

## Abstract

**Purpose:**

Percutaneous epiphysiodesis using transphyseal screws (PETS) was developed as a minimally invasive outpatient procedure to address limb-length discrepancy (LLD) that allowed immediate postoperative weight bearing and was potentially reversible by removing the screws. The aims of our study were to report our results using PETS for LLD and evaluate the accuracy of three growth predictor models.

**Methods:**

Sixteen patients with an average age of 14 years were treated for LLD using PETS. Thirteen patients had screws inserted in a parallel fashion and 3 had crossed screws. We compared the predicted LLD at skeletal maturity using the three growth predictor methods with the actual LLD at skeletal maturity and preoperative LLD with the final LLD at skeletal maturity.

**Results:**

The mean LLD at skeletal maturity between the predicted and final measurements was 0.2 cm using the Green−Anderson method, 1.4 cm using the Moseley method, and −0.1 cm using the Paley method. The mean preoperative LLD of 3.1 cm was corrected to 1.7 cm at skeletal maturity (*p* < 0.001). Six patients complained of pain over the screw heads; however, no patient developed an infection or angular deformity.

**Conclusions:**

The three growth predictor methods predicted the final LLD within an average of 1.4 cm, but there was high variability. Although PETS improved the LLD by a mean of 1.4 cm, we believe the results would have been better if PETS was performed at an earlier skeletal age.

## Introduction

The surgical treatment of limb-length discrepancy (LLD) >2.5 cm was initially described by Phemister using an open technique to create an epiphysiodesis of the longer limb to allow the shorter limb to catch up prior to the end of growth [1]. Since the epiphysiodesis created a permanent growth arrest, an accurate assessment of the patient’s bone age was required to determine the appropriate timing for surgery. Physician concerns about overcorrection often resulted in the procedure being performed later than the ideal recommended time causing an undercorrection. Subsequently, Blount and Clarke developed the technique of epiphyseal stapling by placing three staples on the medial and lateral side spanning the physis to prevent longitudinal growth [2]. The major advantage of the stapling technique was the potential to reverse the growth arrest by removing the staples. Initial problems with staple breakage were addressed by reinforcing the 90-degree angles of the staples, but staple dislodgments and pain over the staples caused many physicians to abandon this method. With the improvement and widespread use of fluoroscopic imaging, physicians developed the technique of percutaneous epiphysiodesis using small curettes, drill bits and dental burs to ablate the physis [3, 4].

In 1998, Métaizeau et al. introduced the technique of percutaneous epiphysiodesis using transphyseal screws (PETS) [5]. The technique involved placing medial and lateral threaded screws across the physis to inhibit growth. The potential advantages of PETS were percutaneous insertion with minimal blood loss, immediate postoperative weight bearing, growth inhibition by two screws would be more stable and comfortable than six staples, and growth inhibition may be reversible by removing the screws, alleviating concerns for overcorrection [6]. Unfortunately, despite these potential advantages, PETS has not gained wide acceptance in North America.

Several reports have documented the success of PETS to inhibit the growth of the longer limb to address LLD [6–9]. There have been complications using PETS including painful screws, placement of the screws too late to achieve the desired effect, as well as angular deformities [8]. Métaizeau et al. reported that the screws began to exert significant growth inhibition within 6 months of insertion, slowing down the distal femoral and upper tibial physes by 68 and 56 %, respectively [5]. Prior studies evaluating PETS used different growth prediction methods including the Green−Anderson growth remaining method [10], the Moseley graph method [11], and the Paley multiplier method [12]. Nouth et al. did not use growth prediction methods and instead focused on the final clinical LLD as their benchmark for success [9].

The aims of our investigation were to evaluate our results using PETS to treat patients with LLD and to evaluate the accuracy of the Green−Anderson method, the Moseley method, and the Paley method in predicting the final radiographic LLD at skeletal maturity.

## Materials and methods

Prior to starting this investigation, we obtained Institutional Review Board (IRB) approval at our medical center. The IRB waived the requirement for informed consent. We reviewed the medical records and radiographs of 16 patients who were treated with PETS for a predicted LLD >2.5 cm at skeletal maturity. Sixteen patients (12 boys and 4 girls) were treated for LLD with PETS in the distal femur and proximal tibia in 15 patients and distal femur in only 1 patient. The average chronologic age at the time of surgery was 14 years (range 11.7−16.1 years) and the average follow-up was 2 years (range 0.7−5.2 years). The etiology of the LLD included 7 congenital, 6 acquired secondary to Legg−Calvé−Perthes disease, fracture causing growth arrest, slipped capital femoral epiphysis, and 3 unknown.

The accuracy of the Green−Anderson growth remaining method, the Moseley graph method, and the Paley multiplier method was determined by comparing the predicted LLD of each method with the actual LLD at skeletal maturity; 95 % confidence interval (CI) was calculated for each method separately. Paired *t*-tests were performed on femoral lengths, tibial lengths, and total limb lengths prior to treatment and at skeletal maturity.

### Surgical technique

**Fig. 1:**
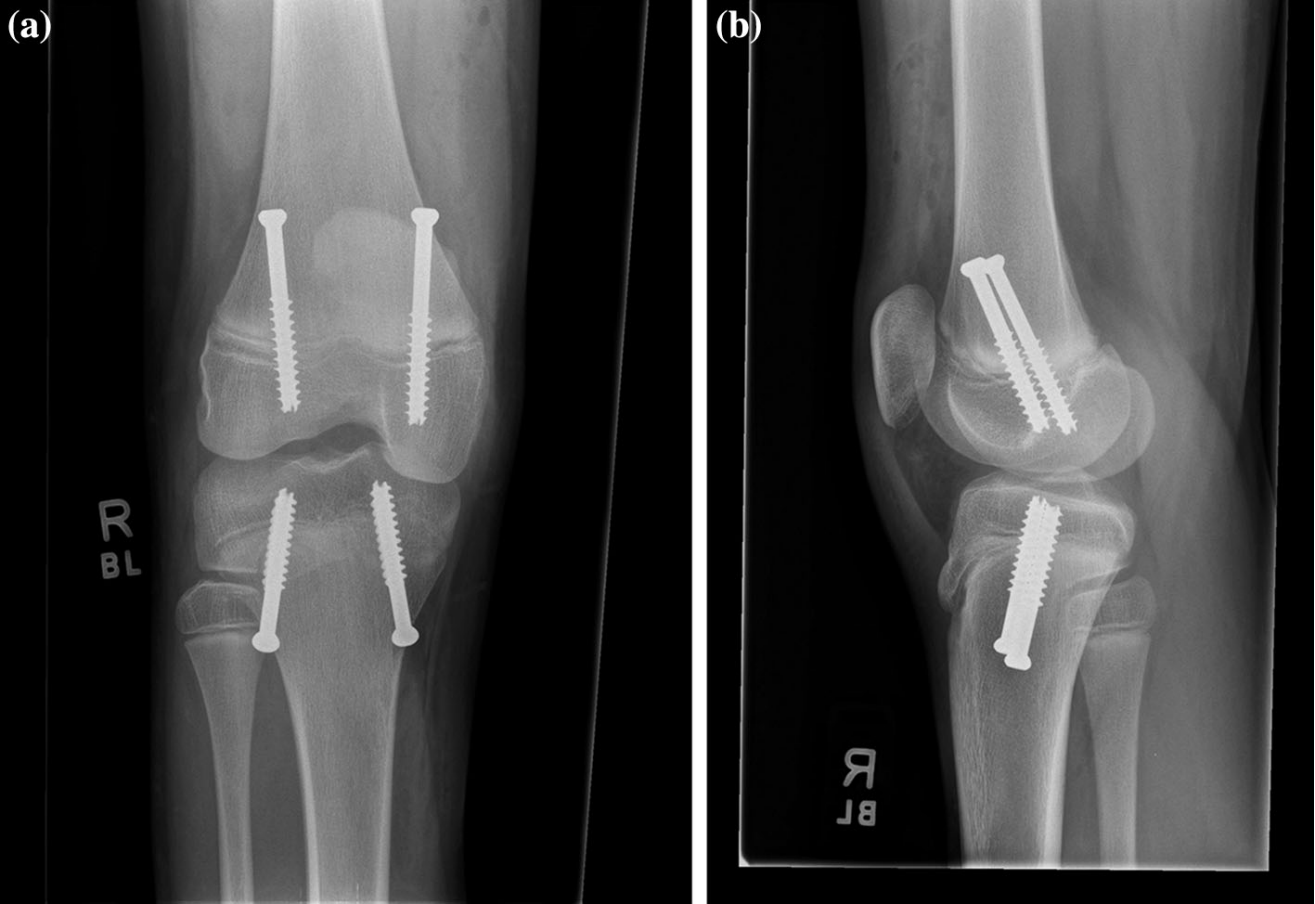
Radiographs demonstrating the parallel technique for PETS. **a** Anteroposterior. **b** Lateral

**Fig. 2:**
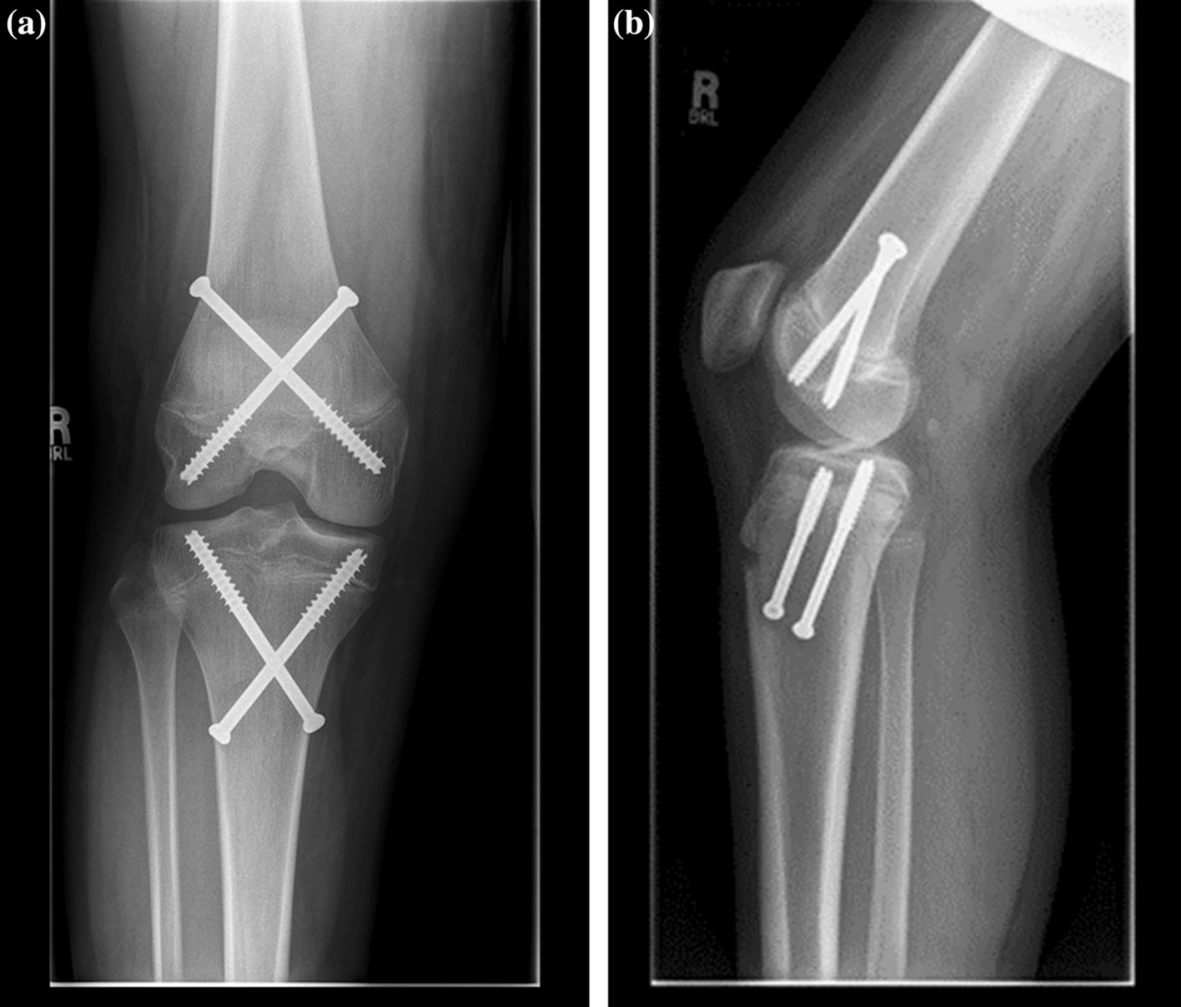
Radiographs demonstrating the crossed technique for PETS. **a** Anteroposterior. **b** Lateral

Patients were placed supine on a standard flat top radiolucent table. Biplane fluoroscopy was used with two C-arms to obtain simultaneous anteroposterior and lateral images of the knee to mitigate accurate screw placement. The entire limb was prepared and draped from the groin to the foot. Fluoroscopy was used to locate the sites for the skin incisions to achieve the most accurate screw trajectory. Screws were inserted in a parallel or crossed fashion according to surgeon preference. When parallel screws were used, on the medial side a guide pin was inserted through a 1-cm incision aiming to cross the physis at the junction of the medial and central one-third in the coronal plane and in the middle one-third of the physis in the sagittal plane. Similarly, on the lateral side a guide pin was inserted through a 1-cm incision aiming to cross the physis at the junction of the lateral and central one-third in the coronal plane and in the middle one-third of the physis in the sagittal plane (Fig. 1). When crossed screws were used, on the medial side a guide pin was inserted through a 1-cm incision aiming to cross the physis at the junction of the lateral and central one-third in the coronal plane and in the middle one-third of the physis in the sagittal plane. Similarly, on the lateral side a guide pin was inserted through a 1-cm incision aiming to cross the physis at the junction of the medial and central one-third in the coronal plane and in the middle one-third of the physis in the sagittal plane (Fig. 2). The guide pins stopped just short of the articular surface and a depth gauge was used to determine screw length. After drilling through the outer cortex under fluoroscopic guidance, 7.3-mm cannulated screws with a 32-mm thread were placed over the guide pins stopping just short of the articular surface. The skin incisions were closed with subcuticular absorbable sutures and covered with a sterile dressing. One patient had parallel screws placed in the distal femur, 12 patients had parallel screws placed in the distal femur and proximal tibia and 3 patients had crossed screws in the distal femur and proximal tibia.

### Data analysis

**Fig. 3:**
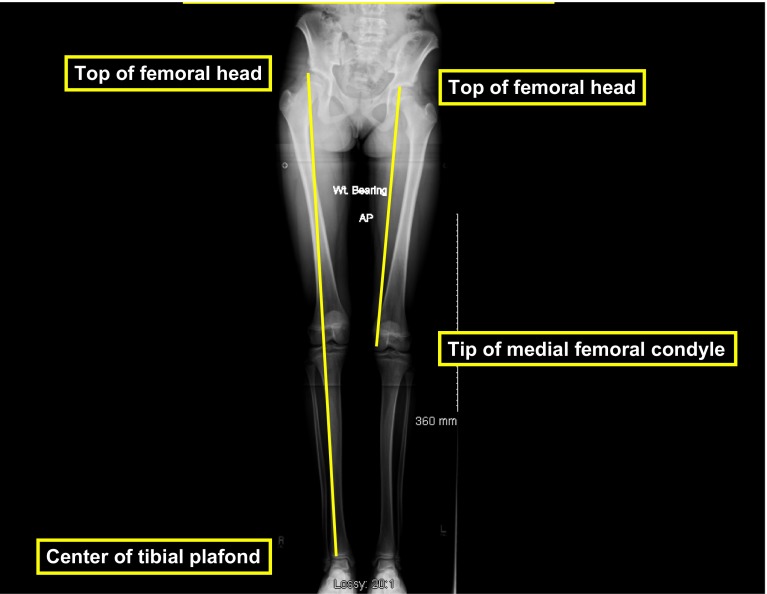
Total limb lengths were measured from the top of the femoral head to the center of the tibial plafond, femoral lengths were measured from the top of the femoral head to the tip of the medial femoral condylar articular surface. Tibial lengths were calculated by subtracting femoral lengths from the total limb lengths

Standing bone length radiographs were obtained to measure total limb lengths, femoral lengths, and tibial lengths of both lower extremities. The radiographic results were reported by the musculoskeletal radiologists and confirmed during outpatient visits by the attending physicians (Fig. 3). Radiographs of the left hand and wrist were performed to determine the skeletal age of the patients from the ‘Radiographic Atlas of Skeletal Development of the Hand and Wrist’ [13]. The skeletal age was reported by the musculoskeletal radiologists and confirmed during outpatient visits by the attending physicians.

The preoperative total limb lengths, femoral lengths, tibial lengths, and LLD prior to surgery were compared with the same parameters at skeletal maturity; a standard paired Student *t*-test was applied to compare the preoperative and postoperative results. The predicted LLD at skeletal maturity was analyzed using the Green−Anderson, Moseley, and Paley methods. The data points collected included predicted limb lengths at maturity without surgery, predicted limb lengths at maturity with surgery, predicted LLD at maturity without surgery, and predicted LLD at maturity with surgery.

### Green−Anderson growth remaining method

When using the Green−Anderson growth remaining charts to calculate the predicted length of the short leg we also calculated and incorporated the growth inhibition rate for the short leg using the formula as described by Lee et al. [14]. Inhibition was defined as the amount of growth of the short limb (S–S’) divided by the amount of growth of the long limb (L-L’) during the same time-interval, subtracted from 1: I = 1 - (S–S’)/(L-L’). The growth remaining in the short leg was thus calculated by multiplying the growth remaining from the Green−Anderson charts times one minus the growth inhibition rate. This allowed for a more accurate assessment of the predicted short leg length without surgery. Green and Anderson reported that 71 % of femoral growth occurs at the distal physis and 57 % of tibial growth occurs at the proximal physis. The predicted femoral, tibial and total limb lengths after surgery were calculated by assuming that PETS would completely halt growth at the physis and that any remaining growth would be due to growth at the proximal femur, distal tibia, or both. Ten patients had sufficient data to be included in the Green−Anderson calculations.

### Moseley graph method

We assumed constant growth in the shorter lower extremity to calculate the projected LLD at maturity from the graph. The date of PETS was plotted on the long leg line based on the most recent measurement of the long leg just prior to surgery. The growth lines on the Moseley graph revealed that surgery on the long leg including a tibial, femoral, or combined epiphysiodesis would have a slope of 72 percent, 63 percent, or 35 percent of normal growth, respectively. Thirteen patients had sufficient data to be included in the Moseley calculations.

### Paley multiplier method

The chronological age of each patient at which the most recent limb-length measurements prior to epiphysiodesis took place was used in conjunction with the multiplier values to determine femoral, tibial and total limb lengths at maturity as well as the predicted LLD. In the event that a patient’s chronological age fell in between the chronological ages for which multiplier values were provided, a more accurate multiplier was calculated using the provided multipliers. For example, a girl with a chronological age of 13 years 6 months would be calculated to have a tibial multiplier of 1.01, given that the provided tibial multipliers for girls at the chronological ages of 13 years and 14 years are 1.02 and 1.00, respectively. Fifteen patients had sufficient data to be included in the Paley calculations.

### Source of funding

No external source of funding was given in support for this study

## Results

**Fig. 4:**
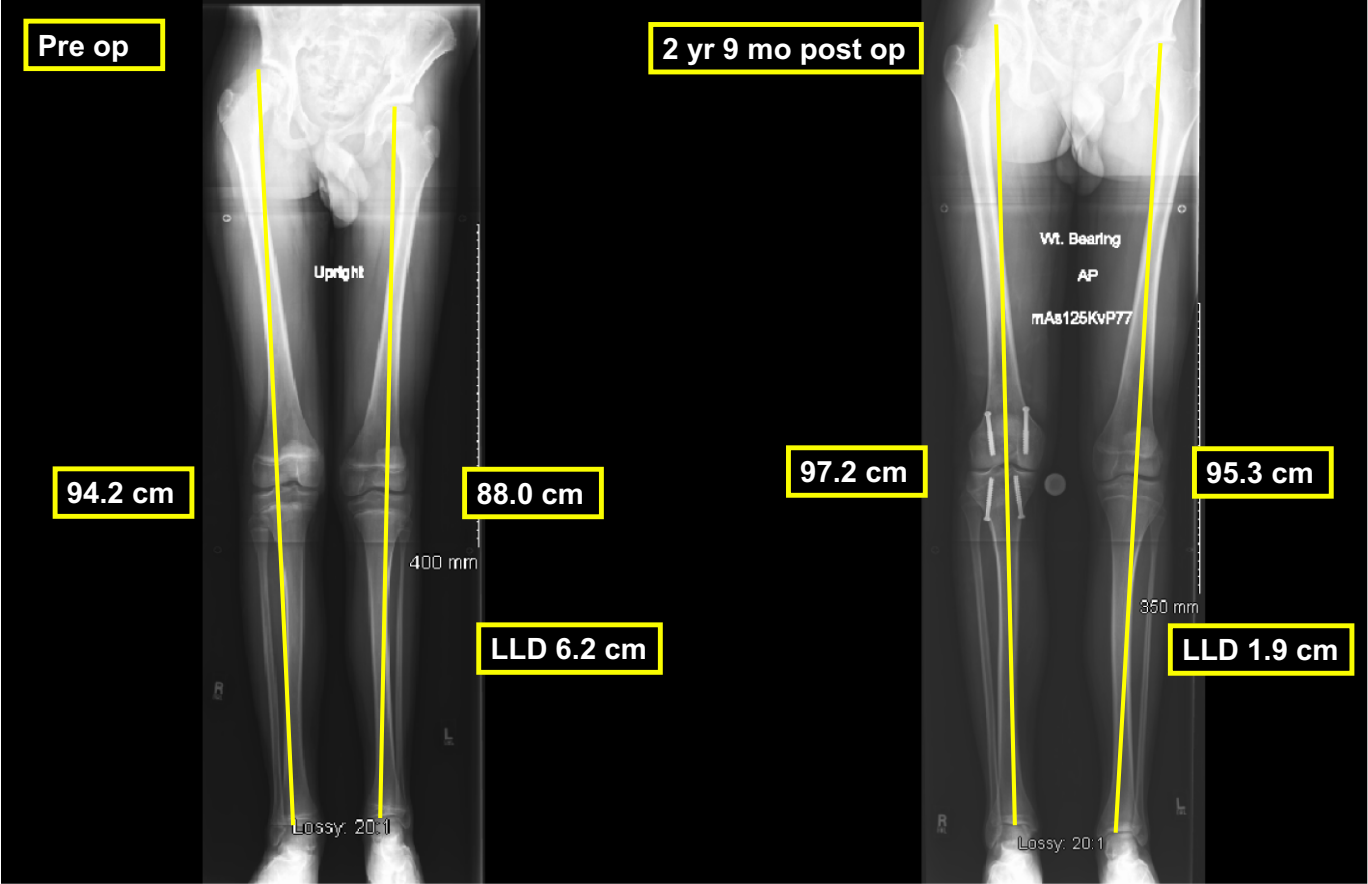
Preoperative bone length study of a 13-year-old boy with LLD of 6.2 cm. We discussed using PETS as his father was 6 feet 3 inches tall and his paternal grandfather was 6 feet 2 inches tall. Two years and 9 months after surgery his LLD was 1.9 cm and his left distal femoral and proximal tibial physes were still open

The PETS technique was successful in decreasing the LLD in 15 of the 16 patients (94 %) patients (Fig. 4). One boy with a preoperative chronologic age of 16 years (bone age 14) showed no improvement in LLD. The average preoperative LLD was 3.1 cm (range 1.3−6.0 cm). The average LLD at maturity was 1.7 cm (range 0.4−3.1 cm) for an average correction of 1.4 cm (range 0.0−4.0 cm; *p* < 0.001) as shown in Table 1.

**Table 1:** Average preoperative and postoperative LLD and the average change at maturity after PETS (*p* < 0.001)

Preoperative			Postoperative			Change	
Mean LLD (cm)	Range (cm)		Mean LLD (cm)	Range (cm)		Mean LLD (cm)	Range (cm)
3.1	1.3–6.0		1.7	0.4–3.1		1.4	0.0–4.0

Six patients (37 %) complained of pain at maturity and had their screws removed. The most common locations of screws that caused pain were the medial distal femur and the medial proximal tibia. The six patients that complained of pain all had parallel screws. There were no complications or difficulties encountered with screw removal although one case required a strong orthopaedic resident. The follow-up radiographs showed no change in the femoral tibial angles in the coronal plane and no evidence of a distal femoral or proximal tibial deformity in the sagittal plane. No patient developed a postoperative infection or other complication.

**Fig. 5:**
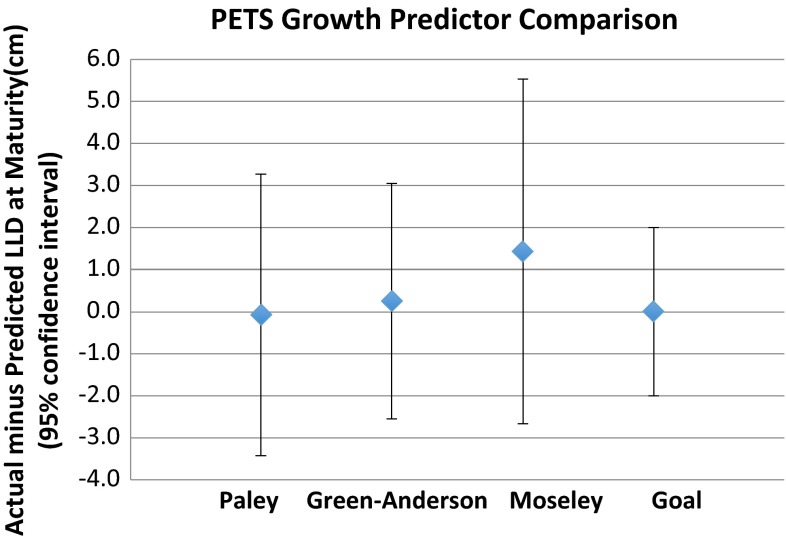
The average difference between actual and predicted measurements of the LLD at maturity was 0.2 cm using the Green−Anderson growth remaining method (95 % CI 2.7 cm), 1.4 cm using the Moseley graph measurements (95 % CI 3.9 cm), and −0.1 cm (95 % CI 3.3 cm) using the Paley multiplier method. The goal was to achieve LLD of 0.0 ± 2.0 cm. Positive values indicate final LLDs were underpredicted and negative values indicate final LLDs were overpredicted

The average difference between actual and predicted measurements of LLD at maturity was 0.2 cm using the Green−Anderson growth remaining method (95 % CI 2.7 cm, 1.4 cm using the Moseley graph measurements (95 % CI 3.9 cm) and −0.1 cm (95 % CI 3.3 cm) using the Paley multiplier method (Fig. 5). Paired *t*-tests showed no significant difference between the three growth prediction methods in predicting the length of the epiphysiodesed limb, the length of shorter limb, or LLD at skeletal maturity.

## Discussion

Phemister [1] developed the concept of performing an epiphysiodesis on the longer limb to address patients with LLDs. The procedure included the resection of a rectangular portion of bone containing the metaphysis and epiphysis, and its reinsertion with ends reversed resulting in a bony bridge. Most physicians also included a simultaneous complete curettage of the physis prior to reinserting the rectangular portion of bone. The disadvantages included irreversibility, considerable postoperative pain requiring hospitalization for pain control and two large scars. These disadvantages led to the development of minimally invasive techniques to achieve an epiphysiodesis. Ramseier et al. [15] reported on the minimally invasive ‘Canale’ technique in 22 patients. Percutaneous epiphysiodesis was performed using a Kirschner wire, a cannulated reamer and a high-speed pneumatic drill. The physis was destroyed using an olive drill working as a reamer and an additional angulated curette. Despite the percutaneous technique, patients still had considerable postoperative discomfort and were restricted from sports for 2 weeks. PETS places two screws across the physis preventing subsequent growth; there is minimal postoperative pain or swelling and the patient can immediately resume activities as tolerated.

The present study confirms the success of the minimally invasive PETS technique performed as an outpatient procedure for treating patients with LLD. Our study also shows that the three growth predictor methods predicted the final LLD within a range of −0.1 to 1.4 cm, although there was large variability in the results. To our knowledge this is the first study to compare the predicted LLD using three common growth predictor methods with the final LLD at maturity after PETS surgery. Compared to other treatment methods, PETS has several advantages including a minimally invasive operative technique, a short learning curve, immediate postoperative weight bearing, and the potential for resumed growth after screw removal [6]. Despite these major advances, questions remain regarding the timing for surgery, placement of screws, types of screws, and reversibility of the growth inhibition by removing the screws.

Métaizeau et al. [5] reported that the screws began to exert a significant growth inhibition effect within 6 months; the delay may be secondary to the time needed for compression to build up across the bony trabeculae. If PETS requires time to inhibit growth, the technique would not be synchronized with the conventional growth predictor methods that base their predictions on an immediate cessation of growth at the time of surgery. As we were concerned about overcorrection, we always recommended the insertion of PETS at or shortly after the time predicted by the conventional growth predictor methods.

Little et al. [16] reviewed 71 epiphysiodeses with adequate orthoroentgenographic and skeletal age data to compare the accuracy of predicting the outcome using the Green−Anderson, Moseley and Menelaus methods. They reported that the three methods showed similar results, but all had limited accuracy. Regardless of the method used, unpredictable results occurred in a proportion of patients. They advocated using the Menelaus method because it is simple, based on chronologic age and proved as accurate as any other method. Lee et al. [14] treated their patients with LLD using a percutaneous epiphysiodesis technique and compared the same three growth prediction methods used in our study. The authors reported that none of the growth predictor methods for calculating LLD at skeletal maturity were accurate although they were clinically effective. In addition, they reported that all the growth predictor methods generated an overcorrected value. In our study, the Green−Anderson and Moseley methods generated undercorrected values while the Paley method generated overcorrected values. These differences may be related to the time after inserting PETS for the screws to begin inhibiting growth and our recommendation to delay surgery to later than the predicted time to prevent overcorrection. Since we believe that growth inhibition by PETS is reversible, we feel the procedure should be performed 12 months prior to the date recommended by the growth predictor methods. Although the three growth predictor methods were able to predict the final LLD between −0.1 and 1.4 cm, the large variability is concerning. Indeed, if PETS is reversible by removing the screws and allowing normal growth to resume as reported in two studies [7, 17], it will resolve the shortcomings of the growth predictor methods, as they will no longer be needed.

The six patients who complained of pain over their screws at maturity had screws placed in a parallel fashion. We are now countersinking the screw heads partially into the cortical bone at the meta-diaphyseal junction to address this concern. Although crossed screws are more technically demanding to achieve precise placement, crossed screws may have less screw head prominence and decrease the risk of irritating the adjacent soft tissues. Song et al. [17] treated 59 patients with PETS using crossed screws and reported no screw-related pain, infection, neurologic injury or hematoma. Three screws broke during attempted removal and the removal was abandoned in 2 others. Five patients (8 %) developed an axial deviation that was attributed to inadequate purchase of the epiphysis on one side in three of the five cases. In our study, no patient developed asymmetric growth that created an axial, coronal or sagittal deformity and we had no problems removing screws. Parallel screws are shorter than crossed screws and can be placed within 10 degrees of perpendicular to the physis in both the anteroposterior and lateral views making them theoretically more mechanically effective to inhibit growth than crossed screws. Since crossed screws are longer than parallel screws, 7.3-mm cannulated screws with a 32-mm thread are placed in a crossed fashion in case they are difficult to remove even though they have reverse-cutting threads. We believe that the ideal screw to address all these shortcomings would be a fully threaded 7.3-mm cannulated stainless steel screw.

Our study has several weaknesses including the small sample size; however, these patients were followed very closely. Not all patients had a bone age study obtained within 6 months of surgery, which limits the use of the Green−Anderson growth remaining method and the Moseley graph method. Not all patients underwent surgery immediately after their preoperative bone length studies, which may have affected the actual LLD before surgery and caused undercorrection of the LLD.

The results of our study demonstrate that PETS is a minimally invasive and safe technique for treating LLD by creating a growth inhibition of the longer limb. The screws can be placed in a parallel or crossed pattern and it may be beneficial to countersink the screw heads, particularly if they are placed in a parallel fashion to decrease the risk of pain from a prominent screw head irritating the soft tissues. Clinicians may use the growth predictor method of choice with the understanding that there may be a large variability in the result, but usually the variability is not clinically significant. We found that the Green−Anderson and Moseley methods tended to underpredict and the Paley method tended to overpredict the final LLD. Several studies have reported that PETS creates delayed growth inhibition and recommend performing PETS between 6 and 12 months earlier than the estimated optimal timing for epiphysiodesis [5, 8, 18]. We believe that PETS is reversible by removing the screws and are now performing the PETS 12 months earlier than the estimated optimal timing for epiphysiodesis. We believe that PETS creates growth inhibition by locking the epiphysis to the metaphysis with screw threads on both sides of the growth plate. Although we have not used fully threaded 7.3-mm cannulated screws, we believe that fully threaded screws would be equally successful and would decrease the problems associated with screw removal.

Further studies are needed to address the timing and reversibility of PETS for correcting LLD. If normal growth resumes after screw removal, the inaccuracies in the growth prediction methods will not be clinically important. However, if there is a rebound effect after screw removal some correction of the LLD will be lost if the screws are removed prior to skeletal maturity. Until more studies are available using PETS for LLD, we believe that it is prudent to use a growth predictor method and to plan the surgery 12 months prior to the estimated optimal timing for epiphysiodesis.
